# Identification of miRNAs regulating MAPT expression and their analysis in plasma of patients with dementia

**DOI:** 10.3389/fnmol.2023.1127163

**Published:** 2023-05-31

**Authors:** Paola Piscopo, Margherita Grasso, Valeria Manzini, Andrea Zeni, Michele Castelluzzo, Francesca Fontana, Giuseppina Talarico, Anna Elisa Castellano, Roberto Rivabene, Alessio Crestini, Giuseppe Bruno, Leonardo Ricci, Michela A. Denti

**Affiliations:** ^1^Department of Neuroscience, Istituto Superiore di Sanità, Rome, Italy; ^2^Department of Cellular, Computational and Integrative Biology, University of Trento, Trento, Italy; ^3^Department of Biology and Biotechnology Charles Darwin, University of Rome “Sapienza”, Rome, Italy; ^4^Department of Physics, University of Trento, Trento, Italy; ^5^Department of Human Neuroscience, University of Rome “Sapienza”, Rome, Italy; ^6^Department of Neurology, IRCCS Neuromed Institute, Pozzilli (IS), Italy

**Keywords:** dementia, MAPT, microRNA-capture affinity technology, microRNA, biomarkers, Alzheimer’s disease, Frontotemporal dementia

## Abstract

**Background:**

Dementia is one of the most common diseases in elderly people and hundreds of thousand new cases per year of Alzheimer’s disease (AD) are estimated. While the recent decade has seen significant advances in the development of novel biomarkers to identify dementias at their early stage, a great effort has been recently made to identify biomarkers able to improve differential diagnosis. However, only few potential candidates, mainly detectable in cerebrospinal fluid (CSF), have been described so far.

**Methods:**

We searched for miRNAs regulating MAPT translation. We employed a capture technology able to find the miRNAs directly bound to the MAPT transcript in cell lines. Afterwards, we evaluated the levels of these miRNAs in plasma samples from FTD (*n* = 42) and AD patients (*n* = 33) and relative healthy controls (HCs) (*n* = 42) by using qRT-PCR.

**Results:**

Firstly, we found all miRNAs that interact with the MAPT transcript. Ten miRNAs have been selected to verify their effect on Tau levels increasing or reducing miRNA levels by using cell transfections with plasmids expressing the miRNAs genes or LNA antagomiRs. Following the results obtained, miR-92a-3p, miR-320a and miR-320b were selected to analyse their levels in plasma samples of patients with FTD and AD respect to HCs. The analysis showed that the miR-92a-1-3p was under-expressed in both AD and FTD compared to HCs. Moreover, miR-320a was upregulated in FTD vs. AD patients, particularly in men when we stratified by sex. Respect to HC, the only difference is showed in men with AD who have reduced levels of this miRNA. Instead, miR-320b is up-regulated in both dementias, but only patients with FTD maintain this trend in both genders.

**Conclusions:**

Our results seem to identify miR-92a-3p and miR-320a as possible good biomarkers to discriminate AD from HC, while miR-320b to discriminate FTD from HC, particularly in males. Combining three miRNAs improves the accuracy only in females, particularly for differential diagnosis (FTD vs. AD) and to distinguish FTD from HC.

## 1. Introduction

The World Health Organization declared that in 2019 over 50 million people in the world have some form of dementia (GBD, 2019 Dementia Forecasting Collaborators, 2022), a condition of chronic and progressive brain function disruption leading to a decline of the cognitive faculties of the person. There are many causes for dementia, including primary neurologic, medical and neuropsychiatric conditions. Among those primary neurodegenerative forms, Frontotemporal Dementia (FTD) and Alzheimer’s disease (AD) share similar clinical symptoms. This fact can often cause misdiagnoses and, consequently, result in inappropriate treatments (Gale et al., 2018). While the recent decade has seen significant advances in the development of novel biomarkers to identify neurodegenerative disorders at their early stage, a great effort has been recently made to identify biomarkers able to improve differential diagnosis of dementias. However, only few potential candidates, mainly detectable in cerebrospinal fluid (CSF), have been described so far (Bjerke and Engelborghs, 2018).

On the other hand, there is an urgent and unmet need of non-invasive and reliable biomarkers to identify patients during early, asymptomatic stages of the disease, when a pharmacological intervention is still possible.

Recently, a novel and promising class of biomarkers for diagnosing and prognosing central nervous system (CNS) diseases has been proposed. Several studies have shown that alterations of the microRNAs (miRNAs), essential components of gene-regulatory networks, play a critical role in the occurrence and development of a variety of neurodegenerative diseases. MiRNAs are a class of small non-coding RNAs, which mainly act as powerful post-transcriptional regulators. In particular, miRNAs target specific mRNAs inducing the repression of translation or their degradation to modulate and fine-tune gene expression levels (Schratt, 2009). They undergo a specific tissutal and temporal distribution, as indicated by several studies on miRNA profiling in different chronic diseases. The alteration of their expression has been associated with almost all cancers and many non-cancer diseases such as AD, multiple sclerosis and heart failure (Leidinger et al., 2013; Keller and Meese, 2016). MiRNAs result long lasting in all human biofluids, because their resistance to degradation. In fact, circulating miRNAs are transported by extracellular vesicles or lipoproteins, (Dorval et al., 2013) and their complexation with proteins, included in the RNA-induced silencing complexes (RISC) (Bartel, 2004) protects them from enzymatic degradation.

Of the about 2,500 mature miRNAs identified in humans (Friedländer et al., 2014), 70% were estimated to be expressed in the nervous system (Adlakha and Saini, 2014), although only a handful of them are expressed in a brain specific or brain-enriched manner. MiRNAs expressed in the CNS mainly regulate neural differentiation, synaptic plasticity and neurite outgrowth (Fehlmann et al., 2016).

MiRNAs are easily detectable in body fluids (plasma, serum, urine, saliva, milk, CSF), using specific simple and sensitive assays. This fact, together with their stability even after a long time period of storage, makes miRNAs good candidates as possible diagnostic and/or prognostic biomarkers in different pathophysiological processes for neurodegenerative disorders and conditions affecting the CNS, particularly in older adults (Grasso et al., 2014, 2015; Piscopo et al., 2016).

Tau is encoded by the MAPT (microtubules associated protein Tau) gene. It interacts with microtubules, modulating their dynamic instability and axonal transport, beyond affects the synaptic function and plasticity. Tau phosphorylation regulates the activity of the proteins and it is directly implicated in pathological processes (Wang and Mandelkow, 2016; Pîrşcoveanu et al., 2017; Naseri et al., 2019). In fact, phosphorylation is able to reduce the Tau tendency to bind microtubules, augmenting its unbounded amount that tend to aggregate (Pîrşcoveanu et al., 2017). Tau has a relevant role in both AD and FTD. Elevated phosphorylation and aggregation of Tau are widely considered pathological hallmarks in AD, while filamentous inclusions of Tau represent a feature in cases of inherited and sporadic FTDs. Moreover, several MAPT mutations were associated to FTD, especially those that alter the ratio between the 6 different Tau isoforms, favoring the more aggregation-prone forms (Wang and Mandelkow, 2016).

The ultimate aim of this study was to identify miRNAs as possible diagnostic molecular biomarkers for dementias. For this propose, we intended to search for miRNAs regulating MAPT translation. Given a mRNA, *in silico* tools to predict miRNA interactors are still not reliable and yield a vast number of candidates. Therefore, we employed a capture technology able to find the miRNAs directly bound to the MAPT transcript in cell lines. Afterwards, we analysed these miRNAs in human samples from FTD and AD patients and relative healthy controls.

## 2. Materials and methods

### 2.1. Cell cultures

Human neuroblastoma Kelly cell line was cultivated in RPMI-1640 medium (Gibco®, Life Technologies) supplemented with 2 mM L-Glutamine, Penicillin/Streptomycin and 10% Fetal Bovine Serum (FBS). All cell cultures were maintained at 37°C in a humidified atmosphere of 5% CO2.

### 2.2. microRNA-capture affinity technology (miR-CATCH)

**Figure 1:**
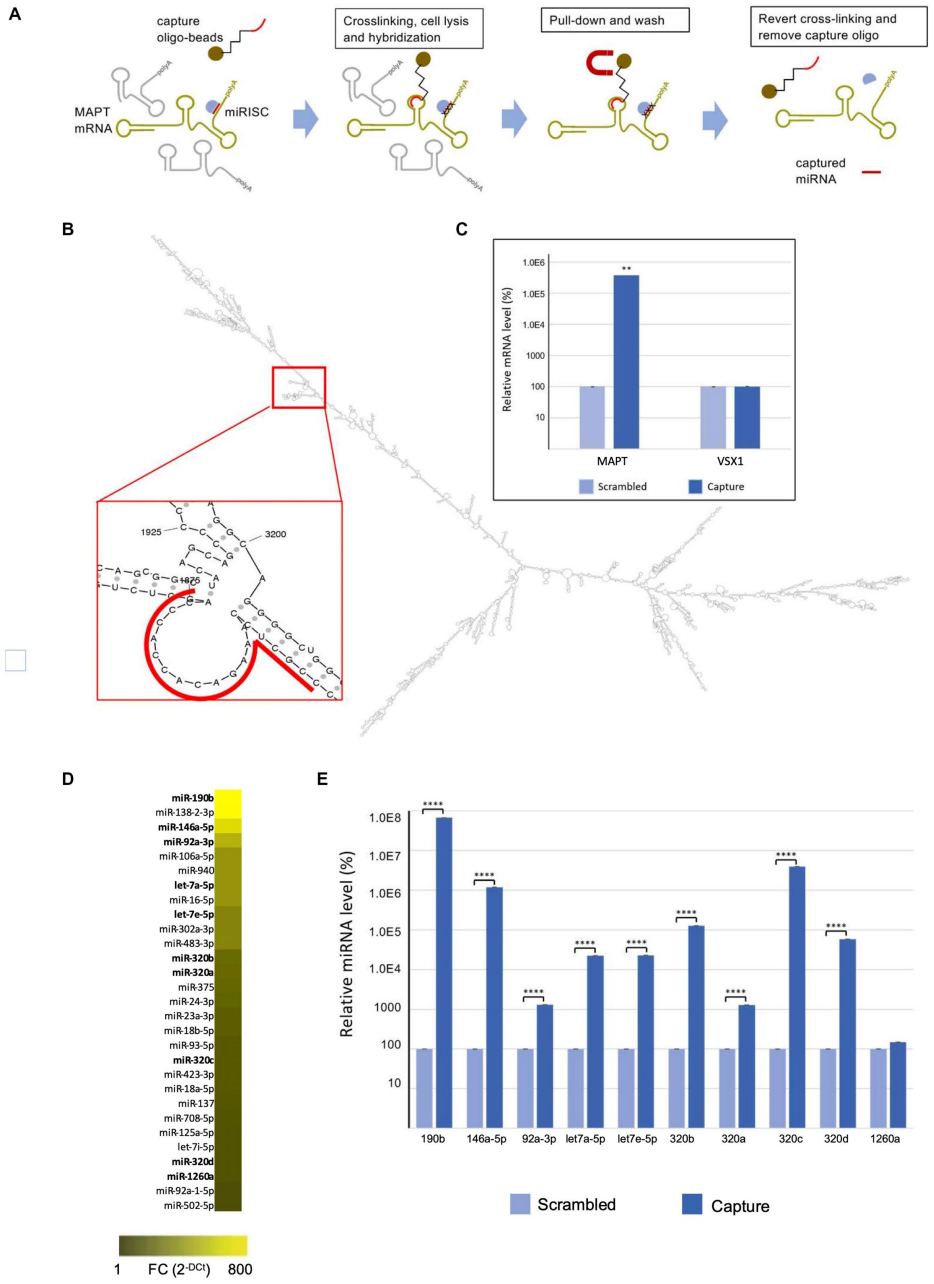
MAPT mRNA miR-CATCH. **(A)** Schematic representation of miR-CATCH (1) active mRNA:miRISC complexes are cross-linked using formaldehyde fixation; (2) cells are lysed; (3) capture oligonucleotide probes complexed with metal beads are hybridized to the target sequence in the mRNA of interest; (4) target mRNAs and the miRISC complexes bound to them are pulled down using magnetic separation; (5) the unbound non-target mRNAs (in grey) are washed away resulting in the enrichment of target mRNA:miRISCs complexes; (6) cross-links are reversed and capture oligonucleotides removed enabling measurement of the enrichment of the target MAPT mRNA and of the captured miRNAs (compared to a scramble capture oligo). **(B)** In the red call-out, the accessible, partially single-stranded region chosen as target for the capture oligonucleotide is shown. The region is located between bases 1855 and 1875 of transcript variant 9 and is present in all variants. **(C)** miR-CATCH was performed in Kelly cells using capture oligonucleotide (Capture; *n* = 3) or scrambled control oligonucleotide (Scrambled; *n* = 3). **(D)** Identification of MAPT mRNA-binding miRNAs. Heat Map showing the miRNAs significantly enriched in MAPT-miR-CATCHed samples. Data are reported as Fold Change (FC = 2-Ct). The ten miRNAs selected for further validation are shown in bold. **(E)** Enrichment of miR-190b, miR-146a-5p, let7a-5p, let7e-5p, miR-320b, miR-320a, miR-320c, miR-320d, miR-92a-3p, and miR-1260a. **** *p* < 0.0001.

The miR-CATCH is based on the pull down of the mRNA of interest together with their naturally bound miRNAs after reversible crosslinking (Figure 1A). The method was performed as described (Zeni et al., 2022).

### 2.3. DNA capture oligonucleotides construction

The MFold web server^1^ was used to model the secondary structure of MAPT mRNA. The thermodynamically most stable structures were analysed with UGENE^2^ to identify the single stranded regions. A specific 5′ biotinylated DNA capture oligonucleotide (5′-GGG TGG TGT CTT TGG AGC GG −3′) was designed against region 1855–1875 of MAPT transcript variant 9 (NCBI NM_001377265.1), region present in all MAPT RNA variants (Figure 1B). The specificity of the capture oligo was analysed with Basic Local Alignment Search Tool (BLAST; http://blast.ncbi.nlm.nih.gov/Blast.cgi), showing a 17 bp complementarity to the *Homo sapiens* visual system homeobox 1 (VSX1) transcript variant 2 mRNA. BLAST A scrambled DNA 5′ biotinylated oligonucleotide (5′-ATA TAT TAG ATT GCG TAT AAT TAG G-3′) was designed as non-specific control for the miR-CATCH. This control oligo does not recognize any human transcript, according to the BLAST analysis performed.

### 2.4. Cross-linking, hybridization, and co-precipitation of complexes

Kelly neuroblastoma cells were crosslinked with 2% paraformaldehyde. After several washes with ice-cold DPBS (Dulbecco’s Phosphate Buffered Saline), cells were lysed and homogenized with glass beads. In parallel, biotinylated capture or scramble oligonucleotides were immobilized on MyOne streptavidin magnetic beads (Dynabeads Magnetic Separation Technology –Thermo Fisher Scientific). Cell lysates were incubated with these beads. After several washes, the beads were incubated at 60°C for 5 min to reverse the interaction between the biotin-labeled DNA: mRNA: miRNA complexes and the magnetic beads. Cross-linked nucleic acids and proteins were incubated for 45 min at 70°C to reverse the cross-linkages. Samples were subsequently used for the following validations.

### 2.5. mRNAs and miRNAs quantification

For the quantification of MAPT transcripts from total RNA, cDNA was synthesized by retrotranscription using RevertAid First Strand cDNA Synthesis Kit (Thermo Scientific®) with oligo (dT) primers, according to the manufacturer’s protocol. Real-Time PCR was performed using Kapa SYBR fast qPCR master mix (Kapa Biosystem®) and primers MAPT_Forward (5’-ACATCCATCATAAACCAGGAGGT-3′) and MAPT_Reverse (5’-TGTCTTGGCTTTGGCGTTCT-3′). PCR reaction (10 μL) contains 0,3 μM of each primer, 5 μL of master mix and 10 ng of cDNA. Then, the levels of mRNAs measured in capture compared to the levels obtained in scramble samples were analysed by the 2-ΔCt method.

The miRNome pull-down in the capture and scramble samples of the miR-CATCH experiments were analysed by reverse-transcription and Real Time PCR (qRT-PCR) using microRNA Ready-to-Use PCR Human Panels (Exiqon). This analysis was performed on 3 independent captures and 3 scramble samples.

For each capture or scramble sample two panels covering 752 human miRNAs were investigated. Data were analysed with Genex software. First inter-plate calibration was performed to compare the results of the first and the second Exiqon panel by using UniSp3 IPC samples present in the plates. Then, N/A values (not expressed miRNA) were substituted with a Ct of 40. Samples with at least 66% of total values were selected, resulting in measurable data for 272 miRNAs. The normalization was performed by the global mean of all miRNAs’ Cts and ∆∆Ct for each selected miRNA.was obtained by using ∆Ct of scramble samples, as controls. The validation of selected miRNAs was performed by TaqMan® microRNA Assay (Applied Biosystems).

### 2.6. microRNA overexpression

The plasmids over-expressing nine miRNAs (let-7a-5p, let-7e-5p, miR-190b, miR-1260a, miR-320a, miR-320b, miR-320c, miR-320d, miR-92a-3p) and miR-181a as a negative control have been generated by cloning a fragment containing the precursor region of miRNAs (pre-miRNA) in the psiUx plasmid, using BglII and XhoI or BglII and KpnI restriction sites. PCR amplification of the fragments was performed on human genomic DNA (#G1471 Promega) with PCR primers detailed in Supplementary Table S1. Plasmids for miR-1260a and miR-181a were available from previous work (Piscopo et al., 2016; Mancini et al., 2021).

For overexpression experiments, 5×105 cells per well were seeded in 24-well dishes and transfected with miRNA-overexpressing plasmids and Lipofectamine3000® (Life Technologies) in a 3:1 ratio. After 48 h proteins were extracted and analysed.

### 2.7. microRNA inhibition

Kelly cells were transfected with miRCURY LNA microRNA Inhibitors (Exiqon) for hsa-miR-92a-1-3p, hsa-miR-320a and hsa-miR-320b and negative Control A (Exiqon).

For inhibition experiments, Kelly cells were transfected at 50–60% confluence with Lipofectamine® 3,000 without P3000 reagent and 12.5 mM/well of miRNA LNA inhibitors. After 48 h proteins were extracted and analysed.

### 2.8. Western blot

20 μg of proteins were separated by 10% SDS-polyacrylamide gel electrophoresis (SDS-PAGE) and transferred on nitrocellulose membrane by using the iBlot® Dry Blotting System (Life Technologies) at 20 V for 7 min. Blots were first blocked with 5% Bovine Serum Albumin (BSA) in PBST (PBS+ 1% Tween 20). Membrane was incubated with a dilute solution of primary rabbit polyclonal anti-Tau Dako and GAPDH (Agilent Technologies® A0024, 1:1000) in 1% powdered milk at room temperature for 1 h or over-night at 4°C and then for 1 h at room temperature with a diluted solution of secondary fluorescent antibody (Abcam®) in 1% of BSA. Membranes were scanned with the LI-COR Odyssey Infrared Imaging System according to the manufacturer’s instructions. Densitometric analysis was performed using Image Studio® and results were normalized by housekeeping GAPDH.

### 2.9. Subject’s recruitment

Patients were recruited from Memory Clinic of “Sapienza” University (Rome, Italy) and from Alzheimer’s Unit of IRCCS Neuromed (Pozzilli, Italy). FTD was diagnosed according to current international diagnostic criteria (Neary et al., 1998; Rascovsky et al., 2011), while the AD diagnosis followed DSM-IV and NINCDS-ADRDA criteria (McKhann et al., 1984). Moreover, all patients underwent standard evaluations including (1) a detailed clinical history recorded from the patients and/or caregivers; (2) an extensive physical exam; (3) neurological examination; (4) an extensive neuropsychological testing including the Mini-Mental State Examination (MMSE); (5) laboratory tests to exclude secondary causes of dementia; (6) brain imaging (magnetic resonance imaging or computerized tomography); and (7) in most cases, 99mTc-HMPAO SPECT or FDG PET scan. All patients were screened for possible mutations in APP, PSEN1, PSEN2, MAPT, GRN, and C9ORF72 genes. Controls were healthy volunteers cognitively normal enrolled among patients’ partners or caregivers. All of them were subjected to a neurological assessment to evaluate cognitive and functional state. The study was approved by ethical committees of both institutes and all the enrolled subjects gave an informed consent.

### 2.10. Samples collection and miRNA quantification

All the procedures were already tested and adapted in our previous works (Piscopo et al., 2018; Grasso et al., 2019). Whole blood was collected from each subject in tubes with EDTA and centrifuged at 2500 rpm, 4°C for 15 min to obtain plasma aliquoted in 250 μL and stored at −80°C. miRNA extraction was performed by the miRNeasy Serum/Plasma Kit (Qiagen), and reverse transcription into cDNA by miScript II RT Kit (Qiagen). cDNA obtained was diluted 1:10 and quantified by qRT-PCR in according to miRCURY LNA SYBR green PCR Kit (Qiagen) manufacturer’s protocol. A critical point in this methodology was to avoid cellular contamination and haemolysis of plasma samples. To check for possible haemolysis contamination of samples, we compared the levels of two miRNAs: hsa-miR-23a-3p, unaffected by haemolysis, and hsa-miR-451a, highly expressed in red blood cells. As reference, we used hsa-miR-93a-5p chosen as the best endogenous control in a previous publication (Grasso et al., 2019). Data of qRT-PCR were expressed as 2-ΔCt.

### 2.11. Statistical analysis

The ΔCt values of miRNA levels was expressed as mean ± standard error. We applied the T-Student test to obtain the *p* values and compare differential expression between two groups (*p* ≤ 0.05). To compare multiple groups, we used ANOVA test and Bonferroni Post-hoc, while Pearson test was used for the correlation analysis. Moreover, we built Receiver Operating Curves (ROCs) and calculated the AUC (Area Under Curve) for each miRNA and combinations of them, to obtain information about biomarkers’ diagnostic accuracy.

## 3. Results

### 3.1. MiR-CATCH identifies miRNAs targeting MAPT mRNA

With the aim of exploring circulating miRNAs to be used as possible biomarkers in FTD and AD differential diagnosis, we decided to look for miRNAs with a functional relevance in these neurodegenerative diseases. We reasoned that Tau protein expression and accumulation has a role in both dementias, and we set out to find which miRNAs directly regulate MAPT expression.

The use of computational analysis to accurately predict putative miRNAs that regulate specific mRNAs remains a challenging process, due to the high false-positive rates of predictions and the use of different algorithms that yield heterogeneous results. To identify the miRNAs binding to the MAPT mRNA, we therefore used an approach called MicroRNA Capture Affinity Technology (miR-CATCH; Figure 1A; Hassan et al., 2013; Zeni et al., 2022).

The method relies on the reversible crosslinking of active mRNA:miRISC complexes in the cells, using formaldehyde. Subsequently, cells were lysed and capture oligonucleotide probes complexed with magnetic beads are hybridized to the target sequence in the mRNA of interest. Target mRNAs and the associated miRISC complexes are then pulled down using magnetic separation and the unbound non-target mRNAs are washed away. Finally, crosslinks are reversed and capture oligonucleotides removed, enabling measurement of the enrichment of the target MAPT mRNA and of the captured miRNAs (compared to a scramble capture oligo).

To design a MAPT-specific capture oligonucleotide, the secondary structure of MAPT mRNA was predicted *in silico* and scanned for single stranded regions, as described in Zeni et al. (2022); (Figure 1B). MAPT mRNA exists in several variants resulting from alternative splicing of exons 2, 3 and 10 (Fontana et al., 2015). As we aimed at pulling down all isoforms, we modeled the longest variant (variant 9 MAPT mRNA, NCBI NM_001377265.1) and chose a target region present in all isoforms. The DNA capture oligonucleotide was designed to bind region 1855–1875 of MAPT mRNA and synthesized to carry a biotin at its 5’end. A scramble 5′-biotinylated oligonucleotide was also designed, to be used as a negative control.

Cells were crosslinked by formaldehyde and subsequently lysed and processed with the biotinylated capture oligonucleotide, in order to isolate MAPT mRNA:miRNA complexes by using streptavidin-coated magnetic beads. In parallel, cells were processed with the scramble biotinylated oligonucleotide. After crosslinking reversion, the eluted samples were analysed by qRT-PCR, which showed about 4,000-fold enrichment of MAPT mRNA in samples processed with the capture oligonucleotide (“capture”), compared to those processed with the scrambled control oligonucleotide (“scramble”) (Figure 1C).

As the capture 20-mer oligonucleotide showed 17-nt complementarity to the VSX1 mRNA in a BLAST analysis (see Materials and Methods), we performed qRT-PCR analyses also with primers specific for the VSX1 mRNA, a transcript not related to MAPT mRNA. As shown in Figure 1C, VSX1 mRNA was not enriched in the capture samples, compared to scramble samples.

Samples were further analysed for the presence of 752 different miRNAs by miRNome qRT-PCR Human panel I + II (Exiqon). Data are available at NCBI’s Gene Expression Omnibus (GEO)^3^ under accession number GSE225256. As shown in Figure 1D and detailed in Supplementary Table S2, 29 miRNAs were significantly enriched in capture samples compared to scramble samples. Out of these, 10 were selected for validation: let-7e-5p, let-7a-5p, miR-92a-3p, miR-146a-5p, miR-190b, miR-320a, miR-320b, miR-320c, miR-320d, miR-1260a (in bold in Figure 1D; Supplementary Table S2).

The relative enrichment of 9 of the selected miRNAs in the capture samples compared to scramble samples was confirmed by qRT-PCR TaqMan single assays (Figure 1E). In descending order of enrichment these miRNAs were: miR-190b (6.7×105-fold), miR-320c (4×104-fold), miR-146a-5p (1.2×104-fold), miR-320b (1.3×103-fold), miR-320d (5.8×102-fold), let-7e-5p (2.3×102-fold), let-7a-5p (2.3×102-fold), miR-320a (1.3×101-fold), miR-92a-3p (1.3×101-fold) (Supplementary Table S3). The enrichment of miR-1260a was not significant when assessed with the TaqMan single assays (Figure 1E; Supplementary Table S3).

### 3.2. miR92a-3p, miR320a and miR-320b regulate tau expression in a neuroblastoma cell line

**Figure 2:**
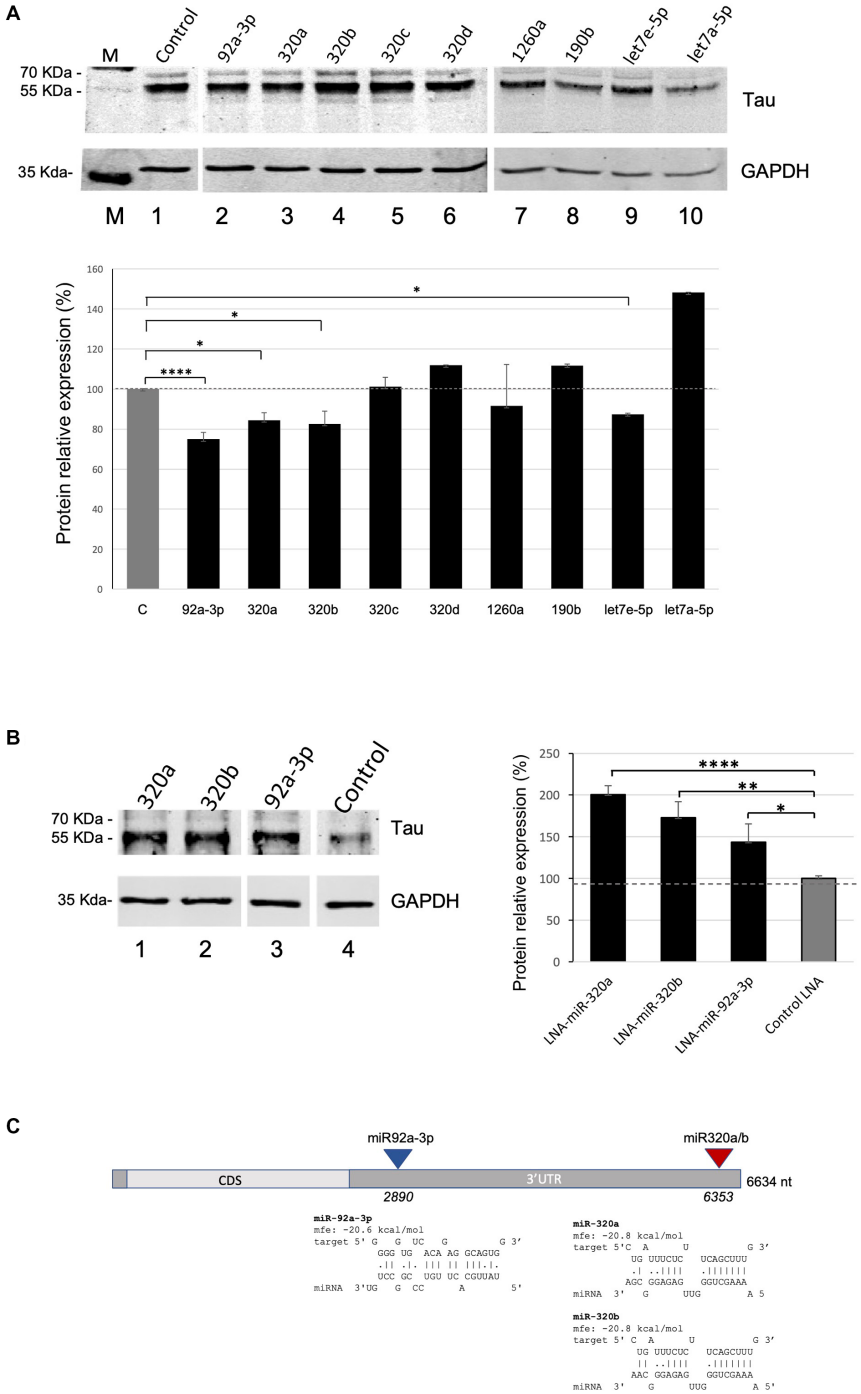
miRNA overexpression and inhibition effects on Tau levels in Kelly cells. **(A)** Effects of the overexpression of miR-92a-3p, miR-320a, miR-320b, miR-320c, miR-320d, miR-1260a, miR-190b, let7a-5p, and let7e-5p on Tau protein levels in Kelly cells. Densitometric analysis from three independent experiments is shown in the lower panel. Data are shown as mean ± S.D. and are compared by t-test for negative control miR (C: Control) versus each miR of interest (value of *p*: *p < 0.05; ****p < 0.0001). **(B)** Effects of the inhibition of miR-320a, miR-320b and miR-92a-3p on Tau protein levels in Kelly cells. Densitometric analysis from three independent experiments is shown in the lower panel. Data are shown as mean ± S.D. and are compared by *t*-test for LNA inhibitor negative control (Control LNA) versus each miR inhibitor of interest (value of *p*: *p < 0.05; **p < 0.01; ****p < 0.0001). **(C)** Predicted binding sites of miR-320a, miR-320b and miR-92a-3p on MAPT mRNA. The miRNAs’ target sequences in MAPT mRNA and their hybridization minimum free energy (mfe) were computed with RNAHybrid. CDS: Coding Sequence; 3’UTR: 3’ Untranslated Region.

We next assessed the functional role of the nine validated miRNAs in regulating Tau expression levels. Western blot experiments were carried out upon transient transfection of the miRNAs-overexpressing plasmids in Kelly neuroblastoma cells (Supplementary Figure S1). They showed that miR-92a-3p, miR-320a, miR-320b, and let7e-5p significatively reduce Tau levels to 75, 84, 83 and 87%, respectively, compared to miR-181a as a control (Figure 2A). No significant variation in Tau levels was observed upon overexpression of miR-190b, miR-320c, miR-320d or miR-1260a, while let7a-5p overexpression unexpectedly significantly increased Tau levels. In this experiment miR-181a was chosen as a negative control since it was not enriched in the capture samples in the above described miR-CATCH experiment, neither was predicted to bind to MAPT mRNA by algorithms commonly used for miRNA targets predictions (e.g., miRDB -www.miRDB.org -or TargetProfiler -mirna.imbb.forth.gr/Targetprofiler).

Kelly cells were then transfected with LNA-based miRNA inhibitors for miR-320a, miR-320b and miR-92a-3p, which induced a significant increase in Tau levels, to 200, 173, 144%, respectively, compared to Control LNA (Figure 2B), as assessed by Western Blots.

To assess the most probable target sites for miR-92a-3p, miR-320a and miR-320b in MAPT mRNA, the miRNA sequences were individually *in silico* hybridized to the best fitting part of the mRNA sequence, and their hybridization minimum free energy (mfe) was computed, using RNAHybrid (Figure 2C).

Predicted binding sites of miR-320a and miR-320b (region 6,353–6,373, part of the 3’UTR) overlapped and their mfe was the same, as expected for the two miRNAs, which differ only by one nucleotide at their 3′ end. MiR-92a-3p was also predicted to bind to the MAPT 3’UTR, in region 2,890–3,011.

### 3.3. Plasma miR-92a-3p, miR-320a, and miR-320b levels differ between patients and healthy controls

After demonstrating that miR-92a-3p, miR-320a and miR-320b regulate MAPT, we investigated their differential expression in plasma samples of patients with AD and FTD and their possible roles as diagnostic biomarkers.

We enrolled a population of 118 subjects, of which 43 healthy controls, 25 females and 18 males (mean age 72.7 ± 7.4), 33 patients with AD, 19 females and 14 males (mean age 69.1 ± 10.4, mean MMSE score 17.27 ± 4.9), 42 with FTD, 28 women and 14 men (mean age 71.1 ± 8.7, mean MMSE score 21.47 ± 5.9), of which 17 subjects displayed the behavioral variant and 17 the semantic variant. All patients were sporadic and no mutations were found in the genes most involved in AD and FTD: APP, PSEN1, PSEN2, MAPT, GRN, and C9ORF72. All the characteristics of the enrolled subjects was summarized in Table 1.

**Table 1:** Characterization of studied population.

Groups	*N*	Sex (M/F)	Onset (mean ± SD)	Age (mean ± SD)	MMSE (mean ± SD)
CT	43	18/25	–	72.74 ± 7.40	–
FTD	42	14/28	66.62 ± 8.31	71.07 ± 8.72	21.47 ± 5.93
bvFTD	17	9/8	67.05 ± 8.09	72.29 ± 8.77	21.67 ± 5.66
svFTD	17	4/13	65.12 ± 8.21	68.88 ± 8.49	22.64 ± 5.69
AD	33	14/19	65.32 ± 7.54	69.15 ± 10.46	16.71 ± 5.73

All values are reported as mean ± standard deviation. bvFTD, behavioral variant FTD; svFTD. semantic variant FTD; SD, Standard Deviation.

**Figure 3:**
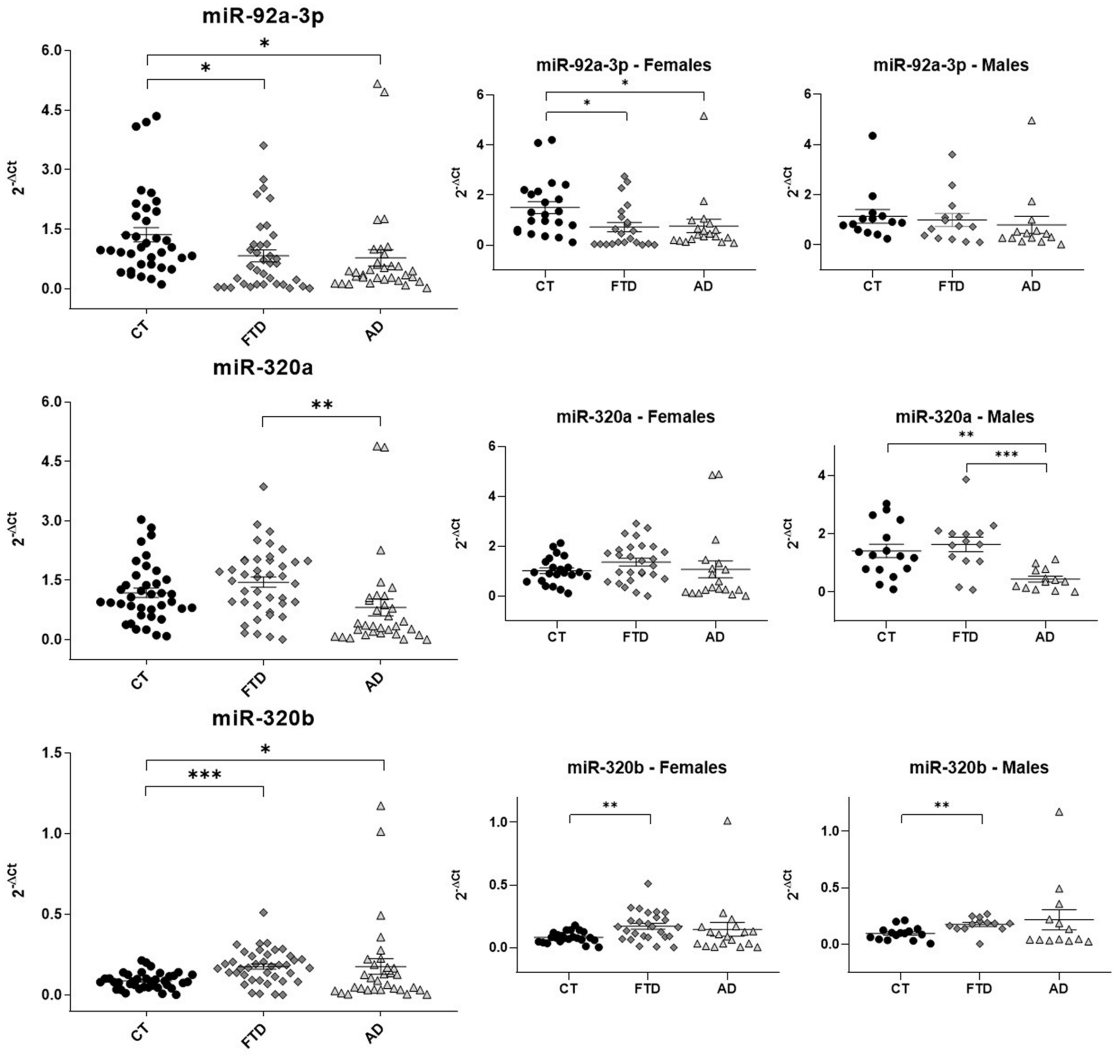
Scatter plots of miRNAs expression levels (2-ΔCt) among CT, FTD and AD in the whole population and in males and females. The data are represented as the mean ± SEM. CT, Controls; FTD, Frontotemporal Dementia; AD, Alzheimer’s Disease; *p ≤ 0.05; **p ≤ 0.01; ***p ≤ 0.001.

Quantitative assays were performed by qRT-PCR (Figure 3), using miR-93a-5p as endogenous control (McKhann et al., 1984; Grasso et al., 2019). Reduced levels of miR-92a-3p were found in both groups of patients compared to healthy controls (FTD 0.83 ± 0.10 versus HC 1.36 ± 0.20, *p* = 0.027; AD 0.78 ± 0.20 versus HC 1.36 ± 0.20, *p* = 0.037). On the contrary, levels of miR-320a did not show differences between patients and controls, but patients with AD had significantly lower levels than patients with FTD (AD 0.81 ± 0.20 versus FTD 1.45 ± 0.10, *p* = 0.009). On the other hand, miR-320b was doubled in both groups of patients respect controls (FTD 0.17 ± 0.02 versus HC 0.09 ± 0.008, *p* < 0.0001; AD 0.17 ± 0.05 versus HC 0.09 ± 0.008, *p* = 0.05).

### 3.4. Circulating miR-92a-3p, miR-320a and miR-320b show sex-related differences

As shown in Figure 3, the downregulation of miR-92a-3p observed in all patients related to controls was confirmed only in women (females: FTD 0.82 ± 0.20 versus HC 1.50 ± 0.20, *p* = 0.02; AD 0.76 ± 0.30 versus HC 1.50 ± 0.20, *p* = 0.045). The analysis of miR-320a by sexes confirmed a lower concentration in AD than FTD in males. Moreover, a significant difference in AD versus healthy controls was also find in men (males: AD 0.44 ± 0.10 versus HC 1.41 ± 0.20, *p* = 0.001; AD 0.44 ± 0.10 versus FTD 1.63 ± 0.20, *p* = 0.0003). On the contrary, we did not observe any inter-group difference in females for miR-320a. Finally, the increase of miR-320b in patients was confirmed only in FTD compared with controls after sex-based stratification (males: FTD 0.17 ± 0.02 versus HC 0.1 ± 0.01, *p* = 0.002; females: FTD 0.17 ± 0.02 versus HC 0.08 ± 0.009, *p* = 0.001).

We then focused on the analysis of FTD sub-phenotypes, but we did not observe any difference among linguistic and behavioral variants. Moreover, possible correlations between miRNAs concentration and age at onset or MMSE score were studied, not finding any significant results.

### 3.5. Roc showed a good diagnostic accuracy for all three miRNAs analysed

**Figure 4:**
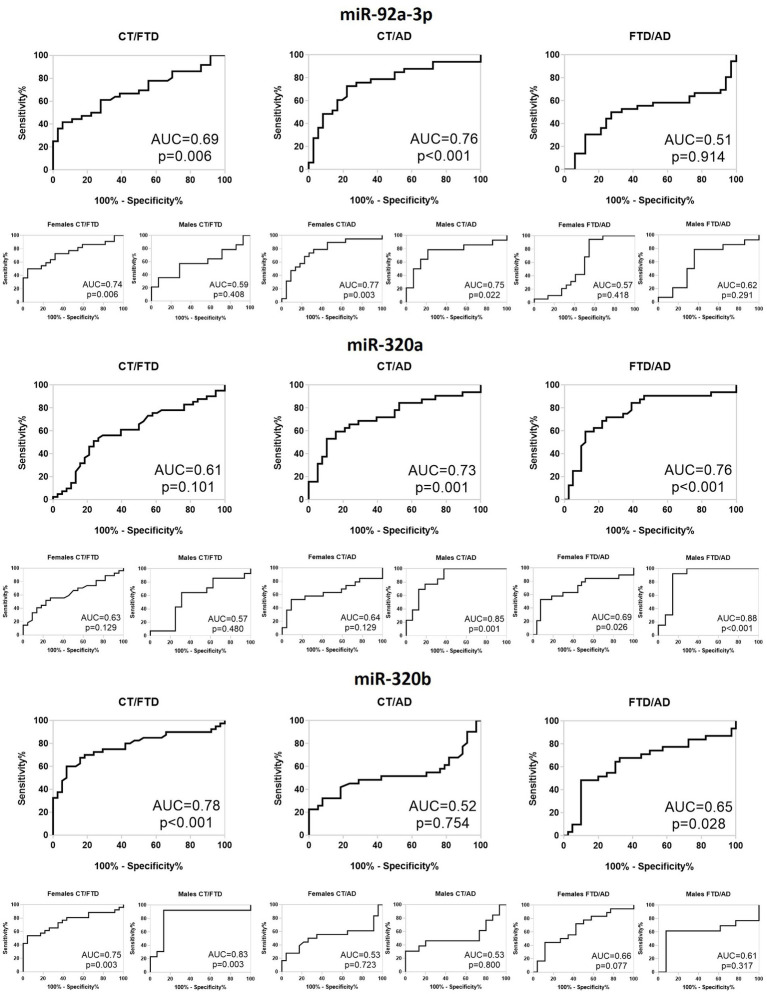
Receiver operating characteristic curve of differentially expressed miR-92a-3p, miR-320a and miR-320b for CT versus FTD, CT versus AD and FTD versus AD in the whole population and in males and females. CT, Controls; FTD, Frontotemporal Dementia; AD, Alzheimer’s Disease; AUC, Area Under Curve.

Lastly, to determine the diagnostic accuracy of the three miRNAs as possible biomarkers, we built ROC curves and calculated the AUC (Figure 4). The miR-92a-3p distinguished healthy controls from AD patients with AUC = 0.76 (*p* = 0.0002) and best accuracy of (60 ± 5)% and 63% sensitivity, both in females (AUC = 0.77; *p* = 0.003; (62 ± 6)% accuracy; 52% sensitivity) and in males (AUC = 0.75; *p* = 0.02; (50 ± 2)% accuracy; 93% sensitivity). It also identifies controls from FTD patients with AUC = 0.69 (*p* = 0.006; (60 ± 5)% accuracy; 26% sensitivity) that reached a value of 0.72 in females (*p* = 0.007; (65 ± 6)% accuracy; 38% sensitivity), while it was not significant in males (AUC = 0.59; *p* = 0.41; (52 ± 8)% accuracy; 29% sensitivity). The same miRNA did not have a good diagnostic accuracy for discrimination of the two patients’ groups nor in the whole population neither after sex-based stratification. Regarding miR-320a, we observed that it discriminates controls from AD with AUC = 0.73 (p = 0.001; (61 ± 4)% accuracy; 84% sensitivity) that reached the value of 0.86 in males after sex-stratification (p = 0.001; (80 ± 6)% accuracy; 63% sensitivity), while its ability to recognize FTD from controls in females was not significant. Interestingly, this miRNA distinguishes FTD and AD with AUC = 0.76 (*p* = 0.0001; (64 ± 4)% accuracy; 78% sensitivity), reaching 0.88 for males (*p* = 0.0008; (77 ± 6)% accuracy; 93% sensitivity) and 69% for females (*p* = 0.03; (61 ± 5)% accuracy; 89% sensitivity). Finally, miR-320b showed a good diagnostic feature in FTD/HC discrimination with an AUC = 0.78 (*p* < 0.0001; (74 ± 4)% accuracy; 92% sensitivity) in the whole population, AUC = 0.84 (p = 0.003; (74 ± 7)% accuracy; 87% sensitivity) in males and AUC = 0.75 (p = 0.003; (75 ± 4)% accuracy; 96% sensitivity) in females, but not in differentiating AD from controls. Furthermore, it was able to differentiate between FTD and AD, but with a low accuracy in whole population (AUC = 0.66; *p* = 0.02; (61 ± 4)% accuracy; 97% sensitivity), and not significant both in males and in females.

### 3.6. The combined measure of miR-92a-3p, miR-320a and miR-320b partially improves diagnostic accuracy

As a further step, we tried several different implementations of a Bayesian classifier specifically designed on miRNAs expression (Ricci et al., 2015; Castelluzzo et al., 2021). The classifier relies on the ΔCt expressions of miR-92a-3p, miR-320a, miR-320b referred, as above, to the normalizer miR-93a-5p. A score is built as a linear combination of the three-ΔCt expressions: Score = ΔCt92a + a * ΔCt320a + b * ΔCt320b, where each one of the two coefficients a and b is varied within the range [−50, 50] with step 0.1. The goal is to find the linear combination that maximizes the diagnostic accuracy. This last parameter is evaluated by assuming the set of scores corresponding to each one of the two, *a priori* determined, diagnostic classes to be normally distributed. Under this hypothesis, the accuracy as well as the threshold to discriminate between the two classes can be analytically calculated. The normality of each optimal score, namely the score corresponding, for each diagnostic case, to the linear combination that maximizes accuracy, was checked via the Shapiro–Wilk normality test.

The sum of miR-92a-3p, miR-320a and miR-320b to discriminate AD and controls provided a Bayesian classifier with a *p* value<0.001, an accuracy of (73 ± 4)% and 55% sensitivity. After sex-stratification, the sum of the three miRNAs to distinguish AD and controls showed (68 ± 6)% and (86 ± 5)% of accuracy with p value 0.02 and 0.001 and sensitivity of 44% and 69%, respectively in females and males (Supplementary Figure S2). Related to FTD vs. HC, the combination of all miRNAs showed an accuracy of (73 ± 5)% in the whole population (*p* < 0.001 and 78% sensitivity), of (77 ± 6)% in females (*p* < 0.001 and 80% sensitivity), and (71 ± 7)% in males (p = 0.02 and 75% sensitivity) (Supplementary Figure S3). Moreover, when we evaluated the capacity to discriminate AD and FTD, we obtained an accuracy of (69 ± 5)%, (71 ± 6)%, and (85 ± 5)%, respectively, in the whole population, females, and males, with p < 0.001 and 91% sensitivity, *p* = 0.003 and 70% sensitivity, and *p* = 0.001 and 92% sensitivity, respectively (Supplementary Figure S4).

## 4. Discussion

In this work, we identified some possible candidates as diagnostic biomarkers of AD and FTD in plasma samples. Firstly, miR-CATCH technique was used to find all those miRNAs that interact with the MAPT transcript. Ten miRNAs have been selected to verify their effect on Tau levels MiR-92a-3p, miR-320a and miR-320b were selected to analyse their levels in plasma samples of patients with FTD and AD respect toHCs. The miR-92a-1-3p is under-expressed in studied pathological conditions compared to controls, a difference that we found even after stratification by gender in females. Moreover, miR-320a appears to be upregulated in FTD patients in comparison to AD also in men when we stratified by sex. It would therefore seem to be useful for differential diagnosis. Respect to HC, the only difference is observed in men with AD who have reduced levels of miR-320a. Instead, miR-320b is up-regulated in both dementias,ROC curve analysis showed that miR-92a-3p and miR-320a could be good biomarkers to discriminate AD from HC, while miR-320b to discriminate FTD from HC particularly in males. Combining three miRNAs improves the accuracy only in females, particularly for differential diagnosis (FTD vs. AD) and to distinguish FTD from HC.

Even if accuracy of CSF AD biomarkers is higher than our miRNAs, we have to consider that these results are obtained by less invasive and easy-to-obtain samples, that also could be useful to monitor the efficacy of a drug by serial analysis during the follow-up and available non only in specialized centers as for PET and CSF analysis.

Mir-92a-3p, miR-320a and miR-320b have already been partially investigated in humans. Denk and colleagues described all three miRNAs as under-expressed in serum of patients of both diseases compared to controls (Denk et al., 2018). Conversely, other studies report the up-regulation of miR-92a-3p in AD (Siedlecki-Wullich et al., 2019), miR-320a and miR-320b in AD and MCI (Nagaraj et al., 2017; Piscopo et al., 2019) versus controls plasma samples. Furthermore, the prediction of the miR-320a and miR-320b targets concerns the APP, BACE1 and MAPT genes (Nagaraj et al., 2017). Our results are in accordance only partially with these reports. This discrepancies in the observations were probably due to the differences in both studied population and in methodology.

Li and his colleagues hypothesized a role for miR-92a-3p on the anxiety state triggered by Alzheimer’s disease. The authors inserted a GFP-Tau-expressing or control transgene into the hippocampus of 2-month-old mice to simulate the increase of brain Tau. Tau increase led the mice to engage in anxious behavior and brain activity was characterized by a decrease in inhibitory signals. After 30 days they found a 60% reduction in extracellular GABA (γ-aminobutyric acid), attributable to the decrease in the vGAT (vesicular GABA transporter) protein, but not in the mRNA, involved in the extracellular transport of GABA. vGAT is a target of miR-92a-3p, which was enriched in tested brains (hippocampus) and in human AD patients. The conclusive hypothesis is that an accumulation of Tau may also lead to an increase in miR-92a-3p in the hippocampus and therefore a reduction in vGAT: GABA is not transported efficiently in the synaptic space and inhibitory signals are lacking. Thus, anxious behavior is amplified (Li et al., 2017).

Mir-92 results expressed in brain and it was described as regulator of neurogenesis in neocortex of mice (Bian et al., 2013; Nowakowski et al., 2013). Interestingly, the inhibition of miR-92 up-regulates the Neogenin protein expression levels in cellular axon guidance, trophectoderm differentiation and neuronal regulation (Wang et al., 2020). It is mainly expressed in glutamatergic neurons (He et al., 2012). An association of mir-92 with structural alterations in hippocampal neurons were also found (Vetere et al., 2014).

Mir-320a and miR-320b were described as relevant in psychiatric disorders (Camkurt et al., 2017). Their presence in the human brain was demonstrated in 2010 by *in situ* hybridation (Nelson et al., 2010). In a systematic review, miR-320 is described as one of the most frequently found miRNAs in the pathways related to neuronal development (Silveira et al., 2020).

Human pathological conditions that have been demonstrated to display a gender disparity are impressive: transmissible and non-transmissible diseases have been investigated in a series of studies aimed at the analysis of the incidence, progression or outcome of very important diseases, such as viral infections, cardiovascular, neurodegenerative, metabolic, respiratory, autoimmune diseases and several form (Buoncervello et al., 2017). Moreover, gender differences in miRNAs’ levels have been described in recent years for some neurodegenerative diseases (Piscopo et al., 2021). Therefore, we chose to extend the analysis by sex finding different results between male and females for miR-92a-3p and miR-320a.

To date, some differences have been observed between males and females in miRNA levels in circulating samples from patients with dementia (Piscopo et al., 2021). MiR-206 is down-expressed in males with FTD and is regulated by hormones in physiological conditions (Grasso et al., 2019). MiR-132 was more upregulated in plasma samples of male patients with Parkinson’s Disease (PD) respect to females. This could be explained by the finding that mir-132 is particularly expressed in the cerebellum of males (Yang et al., 2019). Likewise, miR-29a and miR-29c were described as upregulated in serum of female PD patients, data that could be explained by a major expression of them in the cerebellum of females (Bai et al., 2017). Then, not considering possible differences between males and females in the evaluation of biomarker such as miRNAs, could introduce a sex-bias in the study, with the risk to miss some of these sex-related biomarkers. In conclusion, our results seem to identify miR-92a-3p and miR-320a could be good biomarkers to discriminate AD from HC, while miR-320b to discriminate FTD from HC particularly in males. Combining three miRNAs improves the accuracy only in females, particularly for differential diagnosis (FTD vs. AD) and to distinguish FTD from HC. Considering our results on males and females’ differences, we believe that it is of paramount importance to stratify for sex when performing miRNA analysis aimed at establishing new biomarkers for neurodegenerative diseases. However, to establish the utility of these biomarkers in the differential diagnosis and their possible sex-related role, a larger population should be analysed considering also other types of dementia, such as Levy Body dementia and other neurodegenerative diseases.

## Data availability statement

The data sets generated for this study can be found in the Gene Expression Omnibus, GSE225256 (https://www.ncbi.nlm.nih.gov/geo/query/acc.cgi?acc=GSE225256).

## Ethics statement

The studies involving human participants were reviewed and approved by Sapienza University of Rome (Rome, Italy) and IRCCS Neuromed (Pozzilli, Italy). The patients/participants provided their written informed consent to participate in this study.

## Author contributions

PP: conception, design of the work, acquisition analysis, interpretation of data, and have drafted the work. MG: design of the work, acquisition analysis, and have revised the work. VM: acquisition analysis and have drafted the work. AZ: acquisition analysis. MC: acquisition analysis. FF: acquisition analysis. GT: interpretation of data. AnC: interpretation of data. RR: acquisition analysis and have revised the work. AlC: acquisition analysis. GB: interpretation of data. LR: acquisition analysis and have drafted the work. MAD: conception, design of the work, interpretation of data, and have drafted the work. All authors contributed to the article and approved the submitted version.

## Funding

This work was supported by the Italian Ministry of University and Research (Grant RBFR-0895 DC) and from the University of Trento (MAD) and by CCPP agreement (project no. AB32 CUP I85H20000200005; PP).

## Conflict of interest

PP and RR report being employed by Istituto Superiore di Sanità, GB reports being employed by the University of Rome “Sapienza,” and MAD and LR report being employed by the University of Trento, the three research institutions having a joined patent application pending on the findings described in the present article. PP, MG, AZ, GB, RR, LR, and MAD are co-inventors on this patent and, as such, are entitled to a share of potential royalties.

The remaining authors declare that the research was conducted in the absence of any commercial or financial relationships that could be construed as a potential conflict of interest.

## Publisher’s note

All claims expressed in this article are solely those of the authors and do not necessarily represent those of their affiliated organizations, or those of the publisher, the editors and the reviewers. Any product that may be evaluated in this article, or claim that may be made by its manufacturer, is not guaranteed or endorsed by the publisher.

## Footnotes

^1^http://mfold.rna.albany.edu/?q=mfold

^2^http://ugene.unipro.ru/

^3^https://www.ncbi.nlm.nih.gov/geo
