# Supplementary material for: Identification of miRNAs regulating MAPT expression and their analysis in plasma of patients with dementia
Source: Front Mol Neurosci. 2023 May 31;16:1127163. doi: 10.3389/fnmol.2023.1127163 (PMC10266489; doi:10.3389/fnmol.2023.1127163)

Supplementary Material

Identification of miRNAs regulating MAPT expression and their analysis in plasma of patients with dementia

**^1^*Paola Piscopo, ^2^Margherita Grasso, ^1,3^Valeria Manzini, ^2^Andrea Zeni, ^4^Michele Castelluzzo, ^2^Francesca Fontana, ^5^Giuseppina Talarico, ^6^Anna Elisa Castellano, ^1^Roberto Rivabene, ^1^Alessio Crestini, ^5^Giuseppe Bruno, ^4^Leonardo Ricci, ^2^Michela A. Denti**

^1^Department of Neuroscience, Istituto Superiore di Sanità, Rome, Italy

^2^Department of Cellular, Computational and Integrative Biology, University of Trento, Trento, Italy

^3^ Department of Human Neuroscience, University of Rome “Sapienza”, Rome, Italy

^4^Department of Physics, University of Trento, Povo (TN), Italy

^5^ Department of Human Neuroscience, University of Rome “Sapienza”, Rome, Italy

^6^ Department of Neurology, IRCCS Neuromed Institute, Pozzilli (IS), Italy

*** Correspondence:**Paola Piscopo

[paola.piscopo@iss.it](mailto:paola.piscopo@iss.it)

# Supplementary Figures and Tables

Primer Forward hsa-miR-92a-1-3p 5'-ACTGGGTACCGTGGCCTGCTATTTC-3'

Primer Reverse hsa-miR-92a-1-3p 5'-ACTGctcgagCTGACACGCAACC-3'

Primer Forward hsa-miR-190b 5'-AACTGGGTACCACGAGGACAAGAAAAGG-3'

Primer Reverse hsa-miR-190b 5'-ACTGctcgagATAGTTGCCCGGATGTG-3'

Primer Forward hsa-miR-320a 5'-ACTGGGTACCCTACCCCGAAGCTC-3'

Primer Reverse hsa-miR-320a 5'-ACTGctcgagCCTCTCAGCTCCTC-3'

Primer Forward hsa-miR-320b 5'-ACTGGGTACCGTCCTAGCACCTTTGTT-3'

Primer Reverse hsa-miR-320b 5'-AACTGctcgagGCCATGCTTGATTCCA-3'

Primer Forward hsa-miR-320c 5'-ACTGGGTACCGCCTTTGACCTGTTGA-3'

Primer Reverse hsa-miR-320c 5'-ACTGctcgagGGAAATTCCCTTAGTCTG-3'

Primer Forward hsa-miR-320d 5'-ACTGGGTACCCTCATAAAGCCTCTGAATATTGG-3'

Primer Reverse hsa-miR-320d 5'-ACTGctcgagAGCGAGGCTCTGTCTCAATTA-3'

Primer Forward hsa-let-7a-5p 5'-ACTGGGTACCGCGGATTCAGATAACC-3’

Primer Reverse hsa-let-7a-5p 5'-ACTGctcgagCCCCATCCAGTGTA-3'

Primer Forward hsa-let-7e-5p 5'-ACTGGGTACCACCCGTAGAACCGAC-3’

Primer Reverse hsa-let-7e-5p 5'-ACTGctcgagAGGGAGTCAGAGGATC-3'

**Supplementary Table 1.** PCR primers for plasmids over-expressing let-7a-5p, let-7e-5p, miR-190b, miR-320a, miR-320b, miR-320c, miR-320d, miR-92a-3p

| **miRNA** | **Average Ct**  **Scrambled**  **Control** | **Average Ct**  **MAPT**  **Capture** | **miR-CATCH enrichment**  (2^-ΔCt^) | **p-value** |
| --- | --- | --- | --- | --- |
| **let-7a-5p** | >40.00 | 29.90 | 92.51 | 0.000011 |
| **miR-146a-5p** | >40.00 | 27.28 | 569.06 | 0.000083 |
| **let-7e-5p** | >40.00 | 30.40 | 65.71 | 0.000088 |
| miR-940 | >40.00 | 29.74 | 103.75 | 0.000096 |
| **miR-92a-3p** | >40.00 | 29.16 | 154.68 | 0.000228 |
| miR-483-3p | >40.00 | 30.62 | 56.36 | 0.000280 |
| miR-375 | >40.00 | 31.34 | 34.20 | 0.000350 |
| **miR-320a** | >40.00 | 31.23 | 36.83 | 0.000800 |
| miR-23a-3p | >40.00 | 31.67 | 27.25 | 0.000881 |
| miR-24-3p | >40.00 | 31.50 | 30.68 | 0.001110 |
| miR-16-5p | >40.00 | 30.10 | 80.95 | 0.001129 |
| miR-125a-5p | >40.00 | 32.51 | 15.18 | 0.001155 |
| miR-302a-3p | >40.00 | 30.50 | 61.35 | 0.001538 |
| miR-106a-5p | >40.00 | 29.71 | 106.34 | 0.001603 |
| **miR-320b** | 39.43 | 30.56 | 39.67 | 0.002599 |
| miR-137 | >40.00 | 32.26 | 18.07 | 0.002608 |
| miR-93-5p | >40.00 | 31.93 | 22.74 | 0.002664 |
| miR-18b-5p | 39.52 | 31.22 | 26.70 | 0.002922 |
| miR-18a-5p | >40.00 | 36.62 | 20.98 | 0.003575 |
| miR-502-5p | >40.00 | 32.97 | 11.06 | 0.004164 |
| **miR-320c** | >40.00 | 32.00 | 21.70 | 0.004923 |
| **miR-190b** | >40.00 | 26.81 | 789.05 | 0.005858 |
| miR-708-5p | >40.00 | 32.49 | 15.40 | 0.006068 |
| let-7i-5p | >40.00 | 32.54 | 14.86 | 0.007425 |
| miR-138-2-3p | >40.00 | 26.87 | 759.95 | 0.010554 |
| miR-92a-1-5p | >40.00 | 32.90 | 11.58 | 0.011802 |
| miR-423-3p | >40.00 | 32.04 | 21.06 | 0.014597 |
| **miR-320d** | >40.00 | 32.54 | 14.86 | 0.027151 |
| **miR-1260a** | 38.04 | 30.66 | 14.06 | 0.036365 |

**Supplementary table 2.** MiRNAs enriched more than 10-fold (2^-ΔCt^) with p<0.05. The ten miRNAs selected for validation are shown in bold. Ct>40.00 indicates that the miRNA was not detected in any of the three replicates, and Average Ct was set to 40.00 for further calculations.

| **miRNA** | **Average Ct**  **Scrambled**  **Control** | **Standard**  **Deviation**  **Scrambled Control** | **Average Ct**  **MAPT**  **Capture** | **Standard**  **Deviation**  **MAPT**  **Capture** | **miRNA enrichment**  **TaqMan**  **(2^-ΔCt^)** |
| --- | --- | --- | --- | --- | --- |
| **miR-190b** | >40.00 | 0 | 20.64 | 0.092 | 673784.33 |
| **miR-146a-5p** | 39.01 | 0.056 | 25.47 | 0.600 | 11950.11 |
| **miR-92a-3p** | 32.03 | 0.416 | 28.38 | 0.494 | 13.06 |
| **let-7a-5p** | 37.14 | 0.625 | 29.33 | 0.074 | 225.54 |
| **let-7e-5p** | 33.53 | 0.163 | 25.45 | 0.140 | 230.18 |
| **miR-320b** | 37.96 | 0.451 | 27.64 | 0.095 | 1280.14 |
| **miR-320a** | 33.61 | 0.296 | 29.93 | 0.206 | 12.90 |
| **miR-320c** | 35.02 | 0.072 | 19.75 | 0.203 | 39675.06 |
| **miR-320d** | 37.06 | 0.472 | 27.88 | 0.205 | 586.65 |
| **miR-1260a** | 30.75 | 0.751 | 30.17 | 0.079 | 1.49 |

**Supplementary table 3.** Selected miRNAs in the capture samples after miR-CATCH


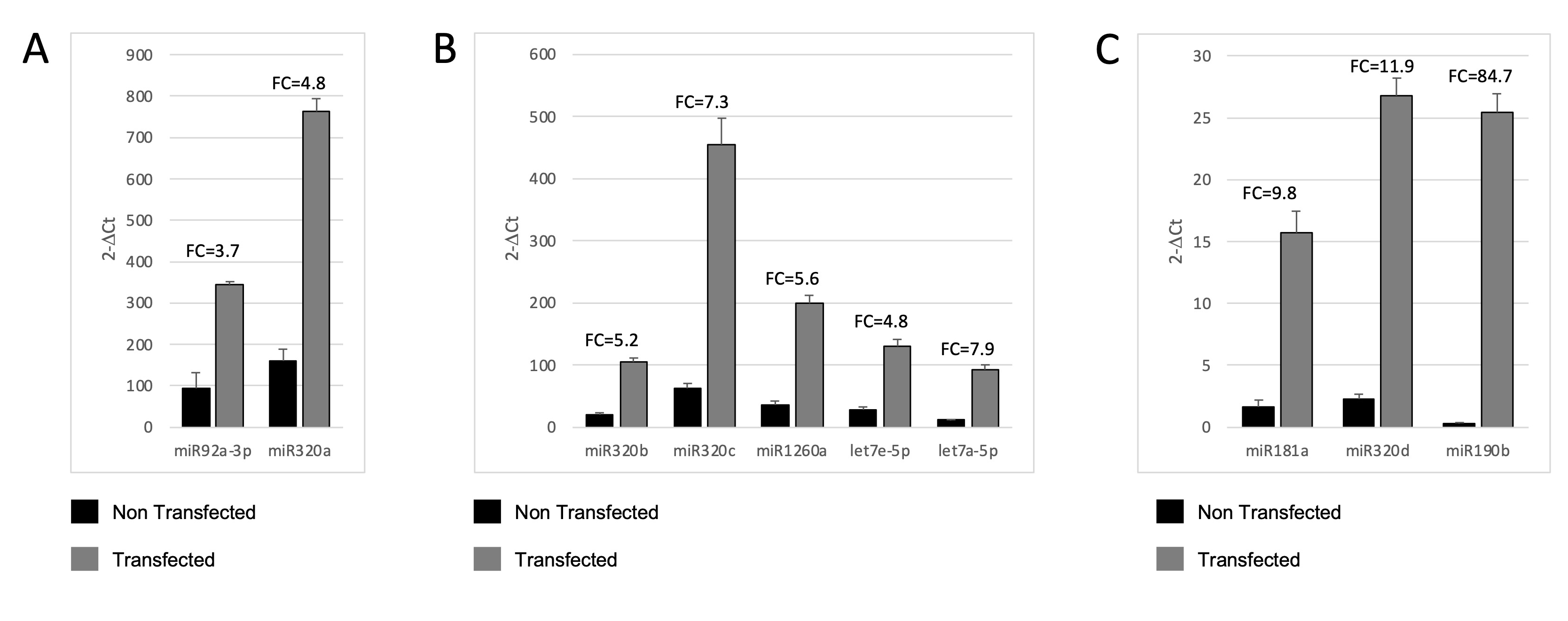


**Supplementary Figure 1**

**Ectopic overexpression of miRNAs in Kelly cells.** The plasmids over-expressing let-7a-5p, let-7e-5p, miR-190b, miR-1260a, miR-320a, miR-320b, miR-320c, miR-320d, miR-92a-3p and miR-181a have been transfected in Kelly cells (Grey bars). 5x10^5^ cells per well were seeded in 24-well dishes and transfected with miRNA-overexpressing plasmids and Lipofectamine3000® (Life Technologies) in a 3:1 ratio. Non-transfected cells were analysed in parallel, to assess the endogenous levels of each miRNA (black bars). After 48 hours proteins were extracted and analysed. miRNAs’ quantification was performed by qRT-PCR using TaqMan® microRNA Assays (Applied Biosystems) as indicated by the manufacturer, and using U6 snRNA Assay for normalization. FC = Fold Change.


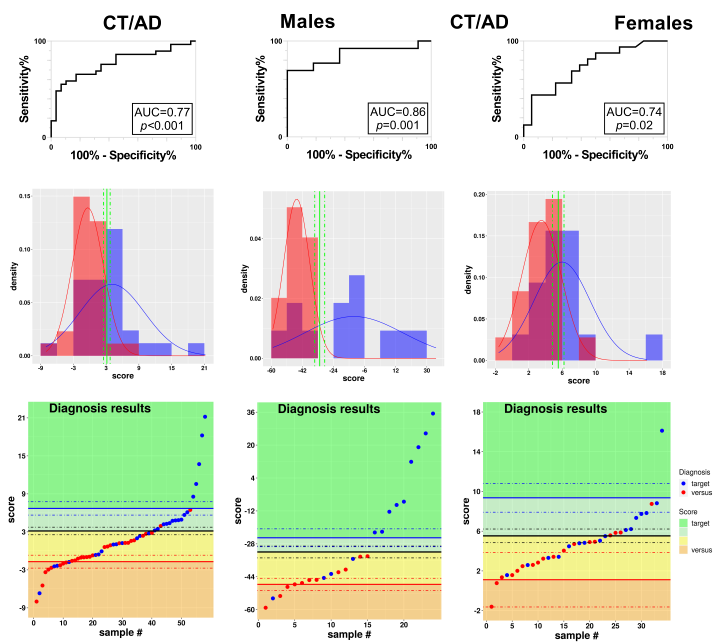


**Supplementary figure 2.** ROC curves (top), histograms (middle) and score plot (bottom) of the combinations of ΔCt of miR-92a, miR-320a and miR-320b, for samples belonging to the target class AD (blue) and class HCs (red). In the histograms, overlapping regions are in magenta. The x-axis represents the score. Each histogram is normalized to the respective set size and thus corresponds to a sample density. The bold lines represent the Gaussian probability densities that fit the data. In the score plot, the bold black lines represents the threshold line for diagnosis while the black dashed lines represent the threshold plus/minus one standard deviation.


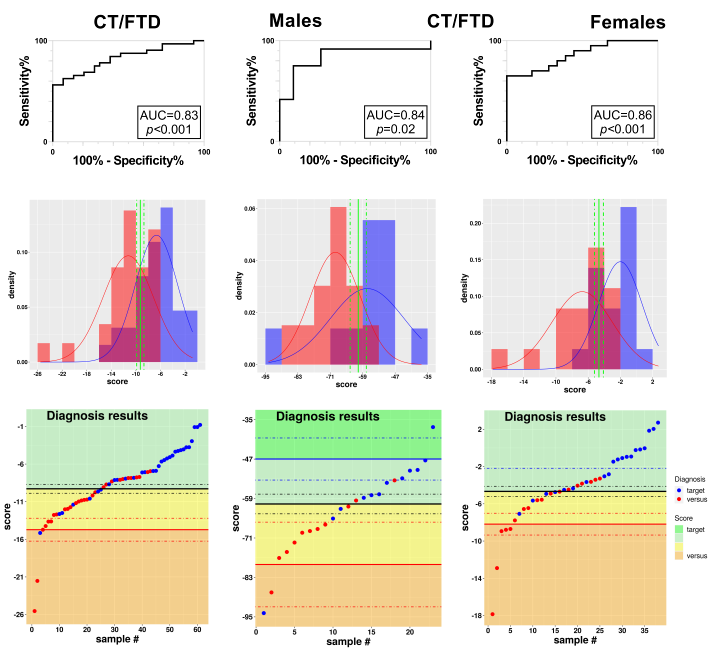


**Supplementary figure 3.** ROC curves (top), histograms (middle) and score plot (bottom) of the combinations of ΔCt of miR-92a, miR-320a and miR-320b, for samples belonging to the target class FTD (blue) and class HCs (red). In the histograms, overlapping regions are in magenta. The x-axis represents the score. Each histogram is normalized to the respective set size and thus corresponds to a sample density. The bold lines represent the Gaussian probability densities that fit the data. In the score plot, the bold black lines represents the threshold line for diagnosis while the black dashed lines represent the threshold plus/minus one standard deviation.

**Supplementary figure 4.** ROC curves (top), histograms (middle) and score plot (bottom) of the combinations of ΔCt of miR-92a, miR-320a and miR-320b, for samples belonging to the target class FTD (blue) and class AD (red). In the histograms, overlapping regions are in magenta. The x-axis represents the score. Each histogram is normalized to the respective set size and thus corresponds to a sample density. The bold lines represent the Gaussian probability densities that fit the data. In the score plot, the bold black lines represents the threshold line for diagnosis while the black dashed lines represent the threshold plus/minus one standard deviation.
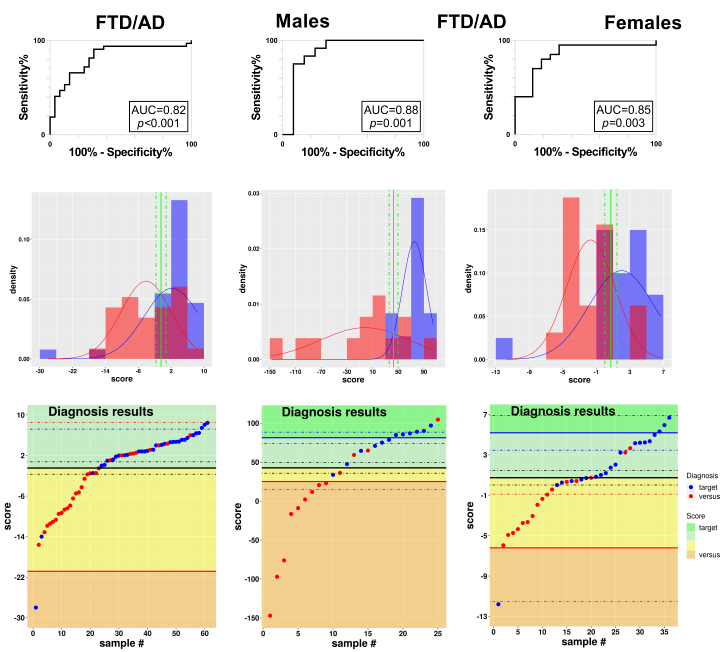

Supplement: Supplementary file 1 [file Data_Sheet_1.docx]
